# Natural Dyeing of Modified Cotton Fabric with Cochineal Dye

**DOI:** 10.3390/molecules27031100

**Published:** 2022-02-07

**Authors:** Ivana Čorak, Iva Brlek, Ana Sutlović, Anita Tarbuk

**Affiliations:** Department of Textile Chemistry and Ecology, University of Zagreb Faculty of Textile Technology, HR-10000 Zagreb, Croatia; ivana.corak@ttf.unizg.hr (I.Č.); ana.sutlovic@ttf.unizg.hr (A.S.); anita.tarbuk@ttf.unizg.hr (A.T.)

**Keywords:** cotton fabric, cationization during mercerization, pre-mordanting, dyeing, natural cochineal dye

## Abstract

Natural dyes are not harmful to the environment owing to their biodegradability. For dye application to textiles, salts are necessary as mordant or electrolytes and make an environmental impact. In this paper, the influence of cationization during mercerization to the dyeing of cotton fabric with natural dye from *Dactylopius coccus* was researched. For this purpose, bleached cotton fabric as well as fabric cationized with Rewin OS was pre-mordanted using iron(II) sulfate heptahydrate (FeSO_4_·7H_2_O) and dyed with natural cochineal dye with and without electrolyte addition. For the characterization of surface changes after cationization, an electrokinetic analysis on SurPASS was performed and compared to pre-mordanting. For determination of dye exhaustion, the analysis of dye solution was performed on a UV/VIS spectrophotometer Cary 50 Solascreen. Spectrophotometric analysis was performed using a Datacolor 850 spectrophotometer, measuring remission ”until tolerance” and the whiteness degree, color parameters, color depth (K/S), and colorfastness of dyed fabric were calculated. Levelness was determined by visual assessment. Cationized cotton fabrics showed better absorption and colorfastness. Pre-mordanting and cationization showed synergism. The electrolytes improved the process of dye absorption. However, when natural dyeing was performed on cotton fabric cationized during mercerization, similar chromacity, uniform color, and colorfastness were achieved with and without electrolyte, resulting in pure purple hue of cochineal. For achieving a violet hue, pre-mordanting with Fe-salt was needed. Therefore, salt can be reduced or even unnecessary, which makes this process of natural dyeing more environmentally friendly.

## 1. Introduction

The application of natural dyes is currently under investigation owing to the multifunctional properties of natural dyes, i.e., inhibition of the growth of some pathogenic bacteria, good protection against UV radiation, and others [1,2,3,4,5]. Natural dyes are easily renewable or derived from waste raw materials of plant origin and are therefore also environmentally friendly [2,3,4,5,6,7,8,9,10]. It is well known that environmental parameters, i.e., chemical (COD) and biochemical (BOD) oxygen demands, are 65% lower than if synthetic dye were used [6,7]. This was confirmed by the authors after natural dyeing with ash bark extract [8]. 

*Dactylopius coccus* is an insect from which natural dye can be extracted. This insect most often lives in tropical and subtropical areas of Mexico and Central America and the northern Andes in South America. It takes 155,000 insects to produce one kilogram of cochineal dye [11,12,13,14]. Extracted dye is named cochineal or carmine due to its chemical structure, which is carminic acid [2,15,16,17]. Cochineal dye—C.I. Natural Red 4, 75470, by Naturex—is chosen for this research because of good regulation status. This dye is a color additive permitted in food and compliant with the purity criteria set by European regulation [18]. Except from meeting strict toxicological parameters for the food industry, one of the main advantages is its ecological acceptability, i.e., good biodegradability. According to the FTIR-ATR spectrum of this specific dye, absorption bands of the main components of carminic acid correspond to anthraquinone compounds. Typical absorption peaks indicate the presence of hydroxyl (–OH), carbonyl (C=O), and carboxyl (–COOH) groups [19,20,21]. 

During the dyeing process, metal salts are used because most of the natural dyestuffs are unable to form strong bonds with fibers. This process is called mordanting and it improves the fastness properties of dyed fabrics. Unfortunately, it can change color properties and the resulting hue, and can have negative environmental impact [7,19,22,23,24]. However, it has been shown that these disadvantages can be avoided. Difference in hue can expand the color palette for creation of specific design, whilst environmental impact can be lowered by optimization of mordant concentration considering the dye and textile material. In last two decades, different concentrations of metal salts were researched from low concentrations of 0.5% owf (over the weight of the fabric) to abnormally high concentrations of 10% [25], 20% [26,27], and even 10–100% owf (5–50 g/L, 1:20 OK) [28,29]. For cochineal dye, Michael et al. suggested 1.6–5% as optimal for cotton depending on the metal salt (KAl(SO_4_)_2_, FeSO_4_, and CuSO_4_) [30], and Brukner et al. optimized KAl(SO_4_)_2_ and CuSO_4_ for wool and synthetic fibers [31]. Sutlović et al. [19,32] researched different mordants for cochineal dye (KAl(SO_4_)_2_·12H_2_O; CuSO_4_ 5H_2_O; FeSO_4_·7H_2_O) in a concentration gradient of 0, 0.5, 3, 5, and 10% owf. It was confirmed that the use of mordant affects the color depth obtained, and that metal concentration of 3–5% owf is satisfactory [19]. The use of higher concentrations of metals can have negative impact to the environment and affect human health [7,19]. From the aspect of the color, more chromatic color shades with color hue in the range 333.77–339.03° were obtained with KAl(SO_4_)_2_, whereas the near achromatic shades with hue range 318.41–332.81° were obtained with FeSO_4_. This is indicating the role of mordant agents in achieving a wider color palette of different shades [19]. Additionally, in previous research [8], the amount of iron and copper after mordanting woolen substrate with FeSO_4_·7H_2_O and CuSO_4_·5H_2_O was studied. The residual amount after mordanting with iron (Fe^2+^) was 6.20 mg/L for 0.5% owf and 6.87 mg/L for 2%, which is within the EU tolerance limit Fe < 10 mg/L. Because of its violet hue and these environmental parameters, FeSO_4_ was used as the mordant in this research.

There are more recent papers concerning the improvement of cotton natural dyeing by plasma, ultrasound, gamma ray irradiation, chitosan, cationic agents, which can help to reduce the addition of mordant and/or electrolyte in the dyeing bath [33,34,35,36,37,38,39,40]. Haji et al. studied plasma treatment and subsequent attachment of chitosan biopolymer for surface functionalization of wool and cotton [37,38], and Peran et al. [36] on wool. Plasma treatment functionalized surface and cationic chitosan contributed to improved dyeability with natural dyes. Application of cationic agents, including full cationization of cotton, leads to salt-free dyeing [36,37,38]. Haddar et al. [33,34] used three commercial cationic agents, Croscolor DRT, Croscolor CF, and Stabifix NCC, for cationization as pretreatment to the dyeing process. Stabifix NCC and Croscolor DRT enhanced exhaustion and fixation of hibiscus extract and fennel leaf extract, respectively. Additionally, the cationization process significantly lowered COD and BOD values [34]. Cationization, a modification with amines and quaternary ammonium compounds, results in a change of fiber surface charge, thus reducing or even eliminating the usage of electrolytes in the dyeing process, and is called “salt free” [33,34,40,41,42,43,44,45,46,47,48,49,50,51,52,53]. The cationization agents and techniques have been intensively researched during the last few decades because of this environmental benefit [48,49,50,51,52]. Most common short-chained compounds are epihalohydrins, 2,3-epoxypropyltrimethyl ammonium chloride (EPTAC), and 3-chloro-2-hydroxypropyltrimethyl ammonium chloride (CHPTAC), which give results within 24 h, but time can be reduced to 5 h [47]. Commercial cationic compounds are usually long-chain compounds with polyammonium bonds. Cationic agents are usually applied by exhaustion and padding as pre- or after-treatment technique, while the cationization during mercerization was recently developed and optimized [48,49,50,51,52,53]. 

Beginning in 2003 [53] and developed by 2009 [51], this modification resulted in new cotton cellulose [41,42]. The process was optimized and it was proven that 5 h is sufficient for epihalohydrines, and only 1 h for long-chain commercial compounds [47,52]. The main difference between techniques of pre- or after-treatment and cationization during mercerization is the levelness after the dyeing process. When the cationization is performed in the after-treatment, it remains on the surface, helping exhaustion and fixation of the dyestuff and anionic auxiliaries, but this treatment blocks surface groups and the color is not leveled. However, if the cationization is performed during the mercerization process, new cellulose is formed, resulting in permanent modification with all benefits of mercerization and cationization [41,42,47,52]. Benefits of mercerization are well known—better strength, gloss, and sorption properties, i.e., dye sorption, as a result of conversion of the crystal lattice from cellulose I to cellulose II accompanied by the change in amorphous region of fiber due to different recrystallization [41,42,52,54,55,56]. Cationization results in the change of the surface charge that ensures further quality improvement, i.e., dye adsorption of 3% direct dye up to 99% without electrolyte [41,42,51] Cationization during mercerization was researched with application of different cationic agents and different characterization methods were performed (FT–IR, SEM, TGA, EKA, surface charge, etc.) [41,42,51,52]. It has been shown that the mercerization is the dominating process, so the changes that can be seen by FT–IR and SEM are contributed to mercerization, and just retained in cationization. If short-chain epihalohydrins were used, the change in cellulose was proven by EKA and TGA. The compounds that were investigated, either short or long-chain, did not have a difference in FT–IR spectrum [51,52]. Only the commercial compound, Rewin OS, if applied during mercerization, showed a weak peak at 1641 cm^−1^, which can be attributed to amine, but it is not representative to perform an analysis [52]. However, electrokinetic analysis (EKA) can be performed since the differences in zeta potential curves are significant and the changes can be well observed. Additionally, it should be noted that cotton fabric cationized during mercerization with cationic reactive polyammonium compound Rewin OS was proven to have positive surface charge and zeta potential, suggesting similar binding to cellulose as epihalohydrins [52]. Since electrokinetics is important for the adsorption of dye anion, in this paper the EKA technique was chosen. Considering the environmental impact of this technique, it is the same as mercerization, which has been a common process in the textile industry for 130 years and has not changed significantly since then. If the cationizating agent was applied during the process, the cationic agent is bonded within the cellulose chains and etherification is complete. Therefore, the modification is permanent and uniform (leveled). In previous research AOX, TOC, and TN value were determined in fabric water extract after the cationization with different agents. Water extract of cotton fabrics before and after modifications indicate that the level of chloride, organic carbon, and nitrogen released by fabrics into the environment is below the EU limits (AOX < 500 μg Cl/L; TOC < 40 mg/L; TN < 2 mg/L) [51].

Compared to synthetic dyes, the color range of natural dyestuffs is rather limited and depends on pretreatment with mordants. For environmental reason, the dyeing of modified cotton cellulose by cationization during mercerization with natural cochineal dye was performed and compared to the usual one with mordant and electrolyte. To reach violet hue, cationized cotton was pre-mordanted as well.

## 2. Materials and Methods

In this research, 100% cotton fabric supplied by Čateks d.o.o. (Čakovec, Croatia) was used. Fabric was plain-woven of mass per unit area 160.8 g/m^2^, yarn density of warp 35.8 threads/cm, and weft 20 threads/cm, scoured and bleached under industrial conditions. 

Cotton fabric was cationized during the mercerization process with cationic reactive polyammonium compound Rewin OS (CHT-Bezema, Montlingen, Switzerland) on a jigger in a two-step procedure at room temperature. Firstly, the mercerization was performed in a bath with 24% NaOH and with 8 g/L Subitol MLF (CHT-Bezema, Switzerland) for 5 passages. Secondly, before fixation in hot water, alkaline cotton fabric was cationized in a bath containing 50 g/L Rewin OS dissolution in water (5 passages), then sealed and left for 1 h at room temperature. Afterward, fixation in hot water, neutralization in 5% acetic acid, and rinsing to neutral was performed. The fabric was air-dried. 

Pre-mordanting of bleached and cationized cotton fabrics was performed in Polycolor, Mathis, LR 1:30, 50 °C, 30 min using iron(II) sulfate heptahydrate (FeSO_4_·7H_2_O) as mordant with a concentration of 5% owf. Fabrics were rinsed with cold water after mordanting.

**Figure 1:**
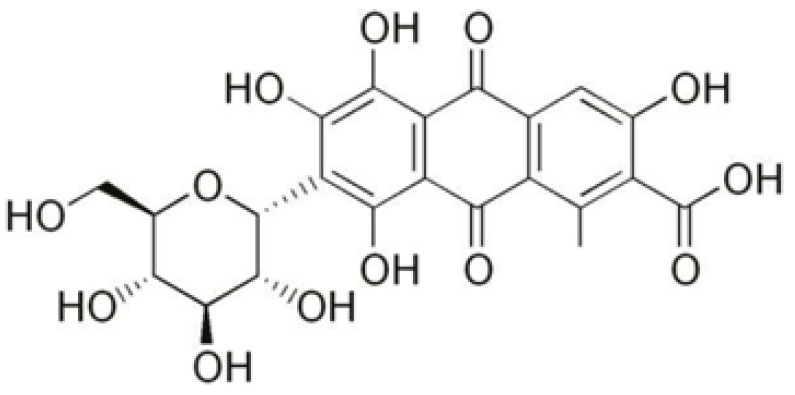
C.I. Natural Red 4, 75470 (Carminic acid).

The dyeing process of cotton fabrics was performed using cochineal dye, C.I. Natural Red 4, 75470, by Naturex, Figure 1. It was performed in Polycolor, Mathis, with a concentration of 6% owf by the exhaustion method with the following parameters: LR 1:30, 60 min, 95 °C, at pH of distilled water (pH 6.5), with and without addition of 40 g/L NaCl as an electrolyte. After the dyeing process, fabrics were washed in cold water, soaped, and again washed. Soaping was performed with Cotoblanc SEL (CHT-Bezema, Switzerland). All samples were air-dried. The dyeing process was performed in two series.

Labels and treatment descriptions used to define the samples are listed in Table 1.

For the characterization of surface changes after cationization and pre-mordanting of cotton fabrics, electrokinetic analysis was performed. The streaming potential was measured with a SurPASS electrokinetic analyzer (Anton Paar GmbH, Graz, Austria) and the electrokinetic potential (zeta, ζ, ZP) was calculated according to the Helmholtz–Smoluchowsky equation [57]. Zeta potential was measured using an adjustable gap cell and was measured as a function of pH of the 1 mmol/L KCl, and the isoelectric point (IEP) was determined.

For the monitoring of dye exhaustion, the analysis of dye solution was performed on a Cary 50 Solascreen UV/VIS spectrophotometer (Varian, Australia). Dye exhaustion (*D_ex_*) was calculated according to the following equation:

*D_ex_* = ((*D*_0_
*− D_B_)/D*_0_) · 100 (1)

where *D_ex_* (%) is exhausted dye, *D*_0_ (g/L) initial dye concentration, and *D_B_* (g/L) the dye concentration in the bath at the end of the process. 

For determination of color fastness, EMPA ECE reference detergent 77 without optical brightener by Testfabrics, Inc. was used. Washing was performed in Polycolor, Mathis, at 40 °C, 40 min with the program Washtest 40 using 2 g/L of detergent.

Spectral characteristics was measured using a Datacolor 850 spectrophotometer according to ISO 105-J01:1997 Textiles—Tests for colour fastness—Part J01: General principles for measurement of surface colour under illuminant D65, 8° standard observer with the specular component excluded and the UV component included. Measurement was performed at random locations on the samples from both series, using the Datacolor Tools computer program and “Measuring until tolerance” command. This means that at least 10 measurements must be made, and the results are accepted when the total color difference between each measurement is less than 0.1 (ΔE* < 0.1). The whiteness degree according to CIE (*W_CIE_*) was calculated automatically according to ISO 105-J02:1997 Textiles—Tests for colour fastness—Part J02: Instrumental assessment of relative whiteness for the cationized and pre-mordanted fabrics. From the results of spectrophotometric characteristics of dyed fabrics, the color depth (*K/S*) and CIEL*a*b* parameters were calculated and compared with the ones after one washing cycle. The total color difference (Δ*E_CMC_*) was calculated using ISO 105-J03:2009 Textiles—Tests for colour fastness—Part J03: Calculation of colour differences. The numerical value of Δ*E_CMC_* within tolerance limits (∆*E_CMC_* ≤ 2) was used for the evaluation [58].

## 3. Results and Discussion 

In this article, the influence of cationization during mercerization to the dyeing of cotton fabric with natural dye from *Dactylopius coccus* was researched. Cotton fabric was cationized during the mercerization process with Rewin OS, and compared to pre-mordanted with FeSO_4_.

**Figure 2:**
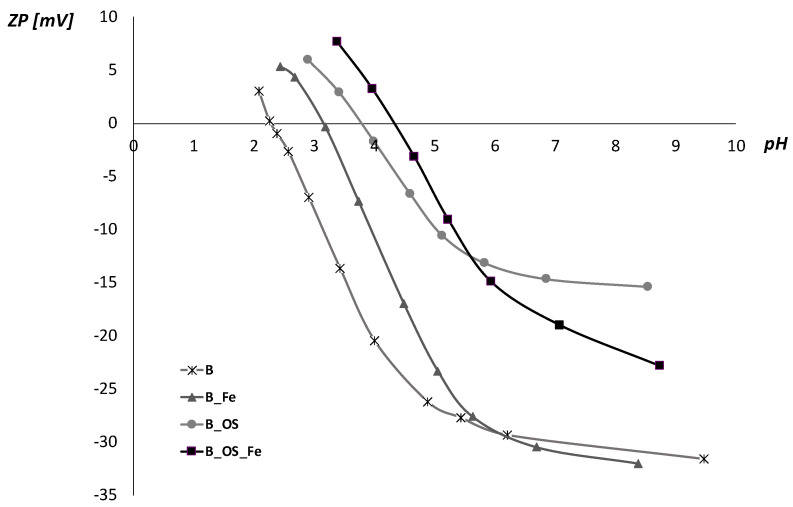
Electrokinetic potential of bleached (B), cationized (_OS), and pre-mordanted (_Fe) cotton fabrics vs. pH of 1 mmol/L KCl.

For the characterization of surface changes after cationization and pre-mordanting of cotton fabrics, electrokinetic analysis was performed and fabric whiteness was determined. Results are presented in Figure 2 and Table 2.

The electrokinetic potential (zeta, ZP) vs. pH of 1 mmol/L KCl was determined on chemically bleached cotton fabrics after cationization with Rewin OS and pre-mordanting with Fe-salt. From the results shown in Figure 2 and Table 2, it can be seen that bleached cotton fabric is negatively charged in the whole pH range due to the presence of carboxyl (–COOH) and hydroxyl (–OH) groups, having IEP at pH 2.4. Carboxyl (–COOH) and hydroxyl (–OH) groups are revealed after scouring and bleaching processes [42,56]. 

Pre-mordanting with iron(II) sulfate heptahydrate slightly increases negative charge in alkaline and neutral media, but in an acidic medium, positive charge of Fe-ions result in lower ZP (−5 mV). Therefore, IEP moves to 3.2 in regard to bleached cotton fabric. 

The notable change in surface charge occurred in the cationization during the mercerization process. In cationized fabrics, -NH_2_ groups are also presented besides –OH and –COOH groups, resulting in higher zeta potential (ZP = −15 mV) for alkaline and neutral media. In an acidic medium, a positive surface charge is achieved, which confirms the binding of cationic compound Rewin OS to the surface sites, moving IEP to pH 3.8. In the case of pre-mordanted cationized fabric, the same phenomenon can be observed as on bleached cotton fabric, i.e., it lowers zeta potential in neutral and alkaline medium, but increase in an acidic one. However, the values of ZP are significantly higher compared to bleached or just pre-mordanted cotton fabrics. Owing to ZP increment, IEP moves to 4.4. 

As it can be seen from the results of whiteness degree presented in Table 2, all pre-treatments have influenced fabric whiteness. Chemically bleached cotton fabric has W_CIE_ = 84.01. Cationization results in a slightly lower whiteness degree, W_CIE_ = 77.81. By adding pre-mordanting agent iron(II) sulfate heptahydrate, the degree of whiteness evidently decreased to W_CIE_ = −31.57 on noncationized cotton fabric and −3.49 on the cationized one. Negative whiteness degree indicates that fabrics changed the color, i.e., it yellowed.

Nevertheless, the results of the electrokinetic analysis indicated that the positive charge of all pre-treated cotton fabrics should result in better dyeing properties. Therefore, the dye exhaustion and color depth were determined.

**Figure 3:**
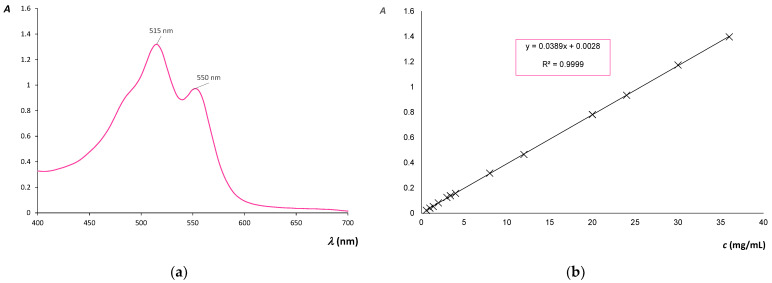
(**a**) Cochineal absorption spectrum. (**b**) Calibration curve for the dye concentration in regard to absorbance.

For the purpose of determination of dye exhaustion, an analysis of dye solution was performed on the UV/VIS spectrophotometer. The absorption spectrum was measured (Figure 3a) and the highest absorption of cochineal dye was determined at 515 nm. Afterward, the solutions for the calibration were measured at 515 nm, and the calibration curve and equation were determined (Figure 3b). This method is based on Lambert–Beer’s law, which provides the function ratio between absorbance (A) and concentration (c).

**Figure 4:**
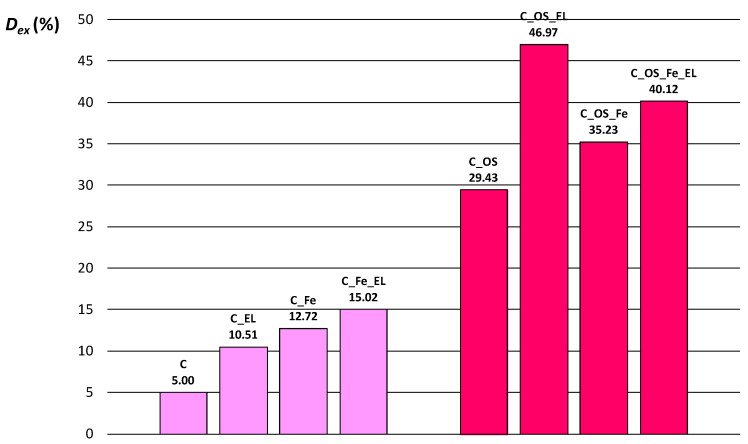
Dye exhaustion of differently pre-treated cotton fabrics.

The calibration curve was used for the calculation of the exhaustion of cochineal dye after the dyeing process. The absorbance of dye solution before and after the dyeing process was measured with the UV/VIS spectrophotometer. The dye exhaustion was calculated taking into account the initial dye concentration and the dye concentration in the bath at the end of the dyeing process. The results are expressed in percent and shown in Figure 4. Bleached, noncationized cotton has exhaustion of natural cochineal dye of only 5%. With addition of electrolyte the exhaustion increases to 10.51%. Pre-mordanting using iron(II) sulfate heptahydrate increases the exhaustion to 12.72%, and 15.02% if electrolyte was added.

It can be seen from Figure 4 that cationized cotton has significantly higher exhaustion compared to noncationized cotton fabrics, regardless of pre-mordanting or electrolyte addition. The dye exhaustion on cationized cotton is 29.43%. The electrolyte addition increase exhaustion to 46.97%. Pre-mordanting of cationized cotton fabric leads to higher exhaustion, 35.23% in regard to 29.43%. However, the exhaustion with electrolyte on pre-mordanted cationized fabric (C_OS_Fe_EL) is not higher than if not pre-mordanted. The highest exhaustion is on cationized fabric with the addition of electrolyte. The main reason for such high dye exhaustion is change of surface charge.

**Figure 5:**
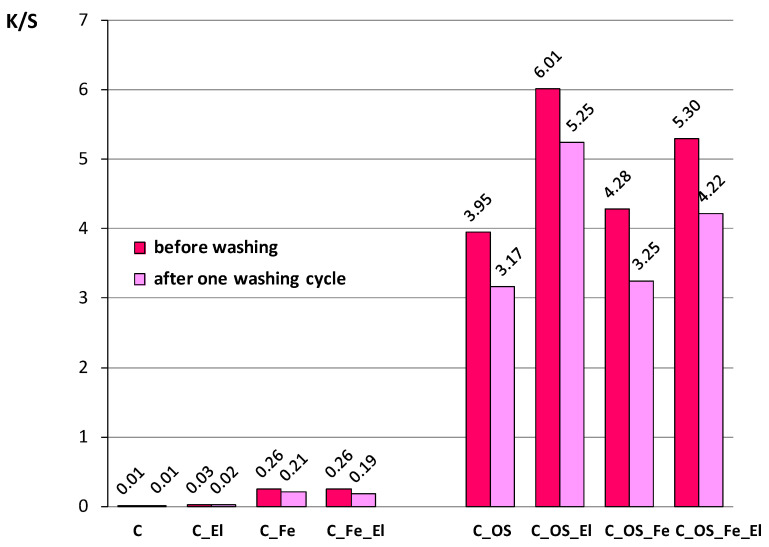
Color depth (K/S) of dyed cotton fabrics before and after one washing cycle (at 520 nm).

**Figure 6:**
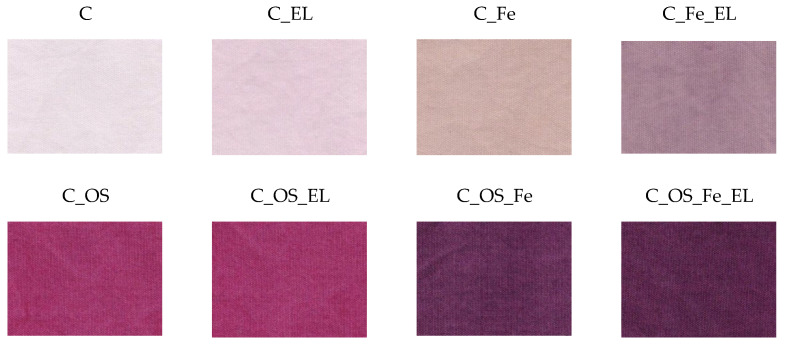
Cotton fabrics dyed with natural cochineal dye.

Spectral analysis was performed from the results of spectrophotometric values after the dyeing process and after one washing cycle for the reason of color fastness. The results of color parameters are shown in Table 3 and Table 4. The color depth coefficient K/S was calculated from remission and presented as K/S maximum at 520 nm in Figure 5. Visual representation of all dyed fabrics is given in Figure 6.

The results of color parameters presented in Table 3 and Table 4 show a color analysis of cotton fabrics pre-treated with different treatments dyed with cochineal dye. Bleached cotton fabric dyed with cochineal dye had a lightness of 94.20 and chroma of 2.19, which correspond to low exhaustion from the dye bath. By adding electrolytes, exhaustion was higher than what resulted in the lightness decreasing and the chroma increasing (C*), but the change was not significant. Pre-mordanting with iron(II) sulfate heptahydrate further improved the absorption of the dye, which led to a decrease in lightness (L*), increased chroma, and a change in color, which is the effect of the binding of iron to the fabric. The C_Fe_EL sample took on a slightly different color, which can be seen in Figure 6. Cationization led to significantly higher exhaustion of dye, which was manifest by a different color hue, a decrease in lightness to 49.17, and an increase in chroma to 36.86. The color hue is in between red and purple. Pre-mordanting of cationized cotton with iron(II) sulfate heptahydrate led to an even greater reduction in lightness and the appearance of a purple color hue. The sample C_OS_EL had the highest chroma, which previously showed the highest exhaustion of the dye from the bath. After the first washing cycle, all samples had a slight increase in lightness, which means that some particles of dye were washed out from the surface. However, it was still significantly higher than without any pre-treatment.

Color depth (K/S) at 520 nm, presented in Figure 5, shows correlation with chroma and dye exhaustion. It is also visible by observation of dye samples (Figure 6). The K/S values obtained very clearly confirm the positive effect of cotton cationization on the natural dye exhaustion and the color depth achieved. Bleached cotton fabric had the smallest K/S value and sample C_OS_EL had the highest. It is visible that cationized cotton fabric has better K/S than noncationized cotton fabrics. By adding electrolytes, K/S changes to a higher level. The reason for this is the change of surface charge in cationization. Except for hydroxyl and carboxyl groups, cationized cotton contains amino groups as well. Therefore, when immersed in water, the amino and carboxyl groups exist in the ionized or zwitterion form.

By adding salt, the diffusion of dye is enhanced. The smallest and most rapidly diffusing ions are quickly adsorbed while the larger and more slowly diffusing dye anions follow, and the kinetics of binding cochineal dye is similar to the one with wool [24,59].

Color levelness describes the uniformity of color in different locations of the fabric. According to visual assessment, supported by Figure 6, it can be seen that cotton fabrics dyed with natural cochineal dye have good levelness regardless of pre-treatment. Small differences in color can be detected by human sight; however, it is quite difficult to quantify color differences. For that reason exactly, spectral remission was measured on random locations “until tolerance”. The observed levelness, especially of cationized cotton fabrics, which exhaust 30–50% of natural dye, confirms that the cationic compound is evenly distributed and trapped between the cellulose chains, so the dyeing process is uniform as well.

**Figure 7:**
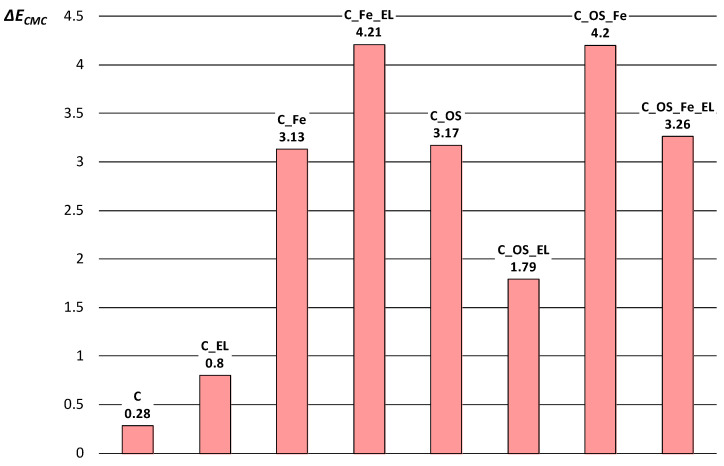
Color fastness of treated fabrics.

Note that the observed phenomena, or K/S values, do not change after one washing cycle. Color fastness analysis was performed by calculating the total color difference (∆*E_CMC_*) between unwashed and washed dyed cotton fabrics. It was calculated after the first washing cycle and presented in Figure 7. From the results obtained, it is visible that the change in color occurred after the first washing cycle, but values of color differences (∆*E_CMC_*) are within the tolerance limits (∆*E_CMC_* ≤ 2) for cotton fabrics that were not pre-mordanted. The reason can be in yellowing after the pre-mordanting process, which significantly changed whiteness degree and therefore results in a changed hue, for the difference when Fe salt was not applied. The smallest Δ*E_CMC_* is on the cotton fabric due to the lowest dye exhaustion. For the fabrics that had high exhaustion the total color difference is higher. Those fabrics exhaust five times more dyestuff, so it is logical that all exhausted natural dye could not be bonded to fibers, and subsequently, it was washed from the fabric and resulted in a higher color difference. It is important to emphasize that the color achieved after the first washing cycle on cationized fabrics was still five times higher than when not cationized. 

## 4. Conclusions

Cationized cotton fabrics adsorb more natural cochineal dye than the noncationized fabrics. When cationized, cotton possesses amino groups together with hydroxyl and carboxyl ones, so the adsorption of anionic dye is very rapid due to attraction. Electrolyte addition contributes to better diffusion of the dye. The best dyeing effect was achieved with cationization and electrolyte addition—the highest absorption rate, the highest chroma, and an acceptable color fastness Δ*E_CMC_*. The results achieved with and without electrolyte are quite similar, so an electrolyte is not necessary. In the case of noncationized fabrics, an addition of electrolyte is important for exhaustion. Pre-mordanting with Fe-salt increases dye exhaustion and color depth, but not much as the cationization process. Pre-mordanting and cationization showed synergism considering dye exhaustion. The exhaustion is higher when the electrolyte was used, but from chromacity achieved and visual assessment it is not necessary. If only cationization is performed, the pure purple hue of cochineal is achieved. If violet hue needs to be achieved, mordanting with Fe-salt is necessary. Thus, when the process of cationization is performed before dyeing with natural dye, salt can be reduced or even unnecessary, making it friendlier for the environment.

## Figures and Tables

**Table 1:** Labels and description of treatments.

Label	Description of Cotton Fabric Treatment
B_	Bleached cotton fabric
_OS_	Cationized cotton with Rewin OS
_Fe_	Pre-mordanting using iron(II) sulfate heptahydrate (FeSO_4_·7H_2_O)
C_	Cotton dyed with cochineal
…_EL	The electrolyte added in the dyeing bath
…_W	One washing cycle after dyeing

**Table 2:** Zeta potential at pH 3.5, 6.5, and 8.5, isoelectric point (IEP), and whiteness degree (W_CIE_) of bleached, cationized, and pre-mordanted cotton fabrics.

Sample	ZP at pH 3.5/mV	ZP at pH 6.5/mV	ZP at pH 8.5/mV	IEP	W_CIE_
B	−15.0	−29.1	−31.3	2.4	84.01
B_Fe	−5.0	−30.0	−32.1	3.2	−31.57
B_OS	2.2	−14.4	−15.0	3.8	77.81
B_OS_Fe	7.3	−17.5	−22.5	4.4	−3.49

**Table 3:** Color parameters of cochineal dyed cotton fabrics.

Sample	L*	a*	b*	C*	h°
C	94.20	0.80	2.04	2.19	68.48
C_EL	92.02	2.85	0.73	2.94	14.42
C_Fe	79.02	6.90	13.78	15.41	63.39
C_Fe_EL	78.80	6.77	11.18	13.07	58.81
C_OS	49.17	36.44	−5.54	36.86	351.35
C_OS_EL	43.73	38.36	−5.45	38.75	351.91
C_OS_Fe	42.28	18.51	−7.48	19.96	338.00
C_OS_Fe_EL	39.77	20.68	−8.20	22.24	338.38

**Table 4:** Color parameters of cochineal dyed cotton fabrics after one washing cycle.

Sample	L*	a*	b*	C*	h°
C_W	94.32	0.56	1.96	2.04	73.95
C_EL_W	92.50	2.20	0.75	2.33	18.85
C_Fe_W	81.29	6.44	15.88	17.14	67.93
C_Fe_EL_W	81.96	5.89	13.83	15.03	66.94
C_OS_W	51.93	34.89	−5.46	35.32	351.11
C_OS_EL_W	45.26	37.44	−5.50	37.84	351.65
C_OS_Fe_W	46.33	18.02	−6.48	19.15	340.23
C_OS_Fe_EL_W	42.97	20.47	−7.56	21.82	339.73

## Data Availability

Data are available in a publicly accessible repository.

## Sample Availability

Samples of the compounds are available from the authors.
